# A study on the availability of national centralized drug procurement in regions with different levels of economic development: an investigation and analysis of 31 provincial-level administrative regions in China

**DOI:** 10.3389/fphar.2026.1652715

**Published:** 2026-01-28

**Authors:** Wei Lu, Yan Qiao, Hongdou Chen, Wei Li, Menglei Wang, Chan Yuan, Qingqing Yang, Yanquan Lin, Yuanyuan Zhao, Lu Ye, Wan Tang, Zhen Yuan

**Affiliations:** 1 Department of Pharmacy, The Affiliated Suqian Hospital of Xuzhou Medical University, Suqian, China; 2 Department of Pharmacy, Nanjing Drum Tower Hospital Group Suqian Hospital, Suqian, China; 3 Distance Learning Support Service Center, Ningxia Polytechnic Ningxia Open University, Yinchuan, China; 4 Drug Prices and Bidding Procurement Division, Suqian City Medical Security Bureau, Suqian, China

**Keywords:** availability, China, national centralized drug procurement, procurement, regions with different economic development levels

## Abstract

**Background:**

China is characterized by significant regional disparities in economic development levels. Accordingly, both the regional implementation effectiveness of the national centralized drug procurement (NCDP) policy and relevant influencing factors urgently require investigation, with the aim of providing evidence to optimize the policy’s implementation.

**Methods:**

The first to fifth batches of NCDP drugs were investigated on the basis of the adjusted standard survey methodology suggested by the World Health Organization/Health Action International. This study has assessed NCDP drug availability across over 900 secondary and tertiary public general hospitals in regions with varying levels of economic development in terms of both the procurement rate and the availability rate.

**Results:**

The availability of these five batches of NCDP drugs in regions with different economic development levels generally followed the pattern that their availability in developed regions is higher than that in moderately developed regions which is also higher than that in less developed regions. A significant difference was observed in the availability of different batches of NCDP drugs (P < 0.05). In developed regions, the average availability rate of each batch was relatively high (from 53.34% to 70.42%), and their procurement rates exceeded 50%. In moderately developed regions, all batches except the fourth exhibited relatively high average availability rates (from 50.08% to 65.14%), and their procurement rates all exceeded 50%. In less developed regions, only two batches (the first batch of “4 + 7” expansion and the second batch) exhibited relatively high average availability rates, and their procurement rates exceeded 50%. The availability of all types of NCDP drugs was also higher in more developed regions.

**Conclusion:**

The implementation of five batches of NCDP drugs has gained initial achievements, but differences persist among regions with varying economic development levels. Accordingly, relevant national departments should optimize the policy implementation mechanism, strengthen the construction of supply chains, and change the inertia of medical behaviour in these regions, aiming to promote the coordinated development of different regions, increase the availability of NCDP drugs and allow people to enjoy policy benefits.

## Introduction

The pharmaceutical procurement model, which has been implemented in China, can be roughly divided into three stages. Before 2000, a decentralized procurement model prevailed with a focus on healthcare institutions. After 2000, provincial drug procurement on the basis of centralized bidding became mainstream; however, the implementation of the “volume-based procurement” approach led to ineffective results. Therefore, efforts were made to improve the centralized drug procurement mechanism and the market-oriented drug price formation mechanism, ease inflated drug prices and the burden of drug expenses on patients, accelerate to import generic versions of drugs, and standardize the supply chain. On 14 November 2018, the Fifth Meeting of the Central Committee for Deepening Reform Comprehensively reviewed and approved the Pilot Plan for National Centralized Drug Procurement, which represented the official start of centralized drug procurement at the national level (Xinhua News Agency, 2018). On 1 January 2019, the General Office of the State Council issued the “Notice on Printing and Distributing the Pilot Plan for National Centralized Drug Procurement and Use of Drugs” (General Office of the State Council of the People’s Republic of China, 2019). This “4 + 7” pilot plan required the four municipalities directly under the central government, i.e., Beijing, Shanghai, Tianjin and Chongqing, as well as seven provincial capitals, i.e., Guangzhou, Shenzhen, Shenyang, Dalian, Xi’an, Chengdu and Xiamen, to implement pilot programmes for volume-based centralized procurement with respect to a total of 25 selected drugs. In September 2019, nine departments, including the National Healthcare Security Administration, issued the “Implementation Opinions on Expanding the Regional Scope of the Pilot Plan for National Centralized Drug Procurement and Use of Drugs” (National Healthcare Security Administration of China, 2019). Based on the 25 generic-name drugs already included in the existing “4 + 7” pilot plan, the document proposed expanding the regional scope of the NCDP pilot, aiming to further reduce patients’ medication cost burden (i.e., out-of-pocket expenses for medicines) and intensify reform and innovation efforts. Notably, this policy adjustment also indirectly promotes drug availability by expanding the coverage of affordable generic drugs across broader regions, which is a core implied objective of centralized procurement. Hence the NCDP model was subsequently extended to cover the whole country. In November 2021, the fifth batch of NCDP drugs was launched in China, which included a total of 218 drug varieties and covered anti-infective drugs, gastrointestinal and metabolism drugs, cardiovascular drugs, anti-cancer drugs and drugs for other common diseases. As a result, their average drug price was reduced by 54%, and particularly the highest reduction rate observed with respect to a single drug was more than 90%; and the five batches of NCDP drugs have saved a total of RMB 250 billion in drug costs (National Healthcare Security Administration of China, 2021; Jing W, 2021). The division of the aforementioned batches of NCDP drugs is not determined arbitrarily, but rather grounded in clear normative bases and a core orientation. The division of these batches is strictly based on the National Drug Centralized Procurement Documents for each batch issued by the National Joint Procurement Office of Drugs, and embodies a dynamic evolutionary framework centred on the core principles of “policy goal guidance, clinical demand orientation, quality bottom-line control, and market structure adaptation” (Sunshine Medical Procurement All-In-One, 2015; General Office of the State Council of the People’s Republic of China, 2019). In terms of policy objectives, the focus has been sharpened on drug varieties that are “clinically necessary, of reliable quality, with excessively high prices, and in large demand”, aiming to achieve “drug price reduction, supply assurance, and innovation promotion”. Regarding clinical needs, priority has been given to drugs for chronic diseases, major diseases, and common diseases, and their dosage form scope has gradually expanded from oral immediate-release dosage forms in the first and second batches to injectables which have accounted for more than half in the fifth batch (Sina Finance, 2021). At the quality and supply level, a unified quality threshold centred on “passing the Generic Drug Quality and Efficacy Consistency Evaluation” (or being an original research drug) has been established, and supplemented by supply chain requirements, under which enterprises should have no GMP (Good Manufacturing Practice) violation records in the past 2 years (Sun, 2025). In terms of market structures, a threshold of an annual procurement volume of no less than RMB 100 million has been set, and the number of competitors has been optimized from no less than three in the initial stage to a maximum of ten shortlisted in the fifth batch, ensuring balanced market efficiency and supply security. The final list of drugs for each batch is determined through a process of “preliminary screening, expert demonstration, and final selection”. The definition of batches in this study is based on their official classification when included in the national centralized procurement.

The NCDP policy requires all public healthcare institutions to participate in the nationally centralized procurement system, and designated private medical facilities and designated retail pharmacies are required under medical insurance agreements to implement the policy in accordance with their contractual management obligations. In 2019, the National Health Commission successively announced two critical directives aimed at enforcing the implementation of the NCDP, namely, “Ensuring the Clinical Allocation and Use of Selected Drugs in the Nationally Centralized Procurement” (National Health Commission of the People’s Republic of China, 2019a) and “Further Improving the Clinical Utilization of NCDP Drugs” (National Health Commission of the People’s Republic of China, 2019b). The implementation of the NCDP for drug allocation and use in healthcare institutions must be enforced to ensure that the reform can benefit more people. To promote the normalized and institutionalized development of the NCDP, in January 2021, the General Office of the State Council of China issued the “Guidelines on Promoting the Regularized and Institutionalized Development of Volume-Based Drug Procurement” (General Office of the State Council of the People’s Republic of China, 2021), which required all public healthcare institutions to collect all NCDP drugs, clarified the scope of coverage, and strengthened safeguard measures in order to ensure the medications accessible to the general public.

The World Health Organization/Health Action International (WHO/HAI) standard survey method is used to evaluate and analyze the accessibility of essential drugs in terms of three dimensions: availability, price level and affordability (World Health Organization and Health Action International, 2008; Chen et al., 2022; Dong et al., 2020). Currently, studies using the WHO/HAI standard survey method to explore the accessibility of drugs have not been limited to essential drugs, and also extended to anti-cancer drugs and drugs negotiated by national medical insurance (Li W. et al., 2024; Ocran Mattila et al., 2023; Varmaghani et al., 2022). The accessibility of NCDP drugs refers to individual opportunity and ability to obtain the drugs covered by the policy and serves as an important criterion for evaluating the implementation of the NCDP system. However, studies have mainly focused on the analysis of the NCDP policy (Wang et al., 2023; Li and Yan, 2021; Liu and Wang, 2021), and a few have addressed the implementation effect of the NCDP policy (Xu et al., 2024; Wen et al., 2023; Zhao and Wu, 2023; Wang et al., 2022; Liu. and Wang, 2022), in which context research on the accessibility of NCDP drugs remains relatively scarce (Zeng et al., 2024; Zou et al., 2023). Moreover, the data sources used in previous empirical studies on this topic have been limited to specific healthcare institutions or specific regions; the varieties of NCDP drugs considered wherein have been limited to a certain batch or a single drug category; and the number of drug varieties covered wherein has been relatively small. In the early stage of this study, by reference to the WHO/HAI standard survey method, the authors first investigated the availability of NCDP drugs included in the first to fifth batches in healthcare institutions at different levels nationwide and revealed significant differences in the availability of NCDP drugs among healthcare institutions (Lu et al., 2023). Subsequently, the results of a study on the accessibility of NCDP proton pump inhibitors in healthcare organizations nationwide indicated that after the implementation of the NCDP policy, the availability of proton pump inhibitors significantly increased, their price level significantly decreased, and their affordability significantly improved (Wang et al., 2024). A typical characteristic of economic development in China is the existence of disparities among regions. These disparities mainly result from such factors as resource conditions, geographic location, and resource allocation capabilities across different regions (Xu et al., 2023). Despite these known disparities, no reports are available regarding the accessibility of NCDP drugs in regions of China with different levels of economic development. Therefore, by reference to the WHO/HAI standard survey method, this study was the first to investigate the availability of NCDP drugs in the first to fifth batches in various regions with different economic development levels in China and to evaluate their accessibility from the perspective of availability. This study can support an understanding of the purchase, procurement and application of NCDP drugs in these regions and provide empirical evidence for evaluating the implementation effect of the NCDP policy.

## Materials and methods

### Data source

The data referenced in this study were obtained from the Shanghai Medical Pharmaceutical All-In-One (SMPA) and the China Medical Economic Information (CMEI) of the Chinese Pharmaceutical Association. The SMPA is a national centralized drug information disclosure platform (Sunshine Medical Procurement All-In-One, 2015), while the CMEI is one of the largest national hospital medication information collection and statistical analysis platforms in China. The latter features a hospital drug use database that covers 29 consecutive years and includes more than 1,500 public hospitals nationwide (Science and Technology Development Center of Chinese Pharmaceutical Association, 1996).

### Sample representativeness and data selection

The targets of this study were the first to the fifth batches of NCDP drugs, which covered a total of 218 varieties. For the procurement cycle of the NCDP, the first batch of the “4 + 7” pilot plan has a 1-year cycle, while the cycles of all other batches are determined based on the number of winning bidding enterprises (i.e., enterprises that successfully won the bidding for the procurement of the target drugs), ranging from one to 3 years. In principle, when the number of winning bidding enterprises is small, the procurement cycle is relatively shorter; and when the number is large, the cycle is relatively longer. The list of the five batches of NCDP drugs was taken from the SMPA. The quantity of drugs was calculated by reference to the total number of NCDP drugs during the procurement cycle following the implementation of each batch. The specific information is presented in Supplementary Table S1. In light of the *per capita* gross domestic product (GDP) of each provincial administrative region in China (National Bureau of Statistics of China, 2022) and the discussion on China’s economic regional development presented by Wei et al. (2020), the levels of economic development in China are divided into developed regions, moderately developed regions and less developed regions. The distribution of each provincial-level administrative region is indicated in Supplementary Table S2. Based on the information regarding the drug name, dosage form, specifications and packaging, manufacturer, winning bid price, and designated supply province (region) in the selected catalogues of five batches of the NCDP drugs, the authors extracted purchase patterns pertaining to secondary and tertiary public general hospitals in 31 provincial administrative regions (excluding Taiwan, Hong Kong, and Macao) during the purchase cycle from the CMEI. The characteristics of the hospitals in the samples are presented in Supplementary Table S3 and Supplementary Figure S1. For the classification of NCDP drugs included in this study, the five batches were categorized based on the Anatomical Therapeutic Chemical (ATC) classification system and the “2022 National Reimbursement Drug List for Basic Medical Insurance, Work-Related Injury Insurance and Maternity Insurance (Western Medicine Catalogue)”. The distribution of the number of NCDP drug varieties is shown in Supplementary Table S4.

The hospital samples in this study were derived from the fixed medical institution sampling frame monitored by the CMEI, which covers 31 provincial administrative regions in China, ensuring the inclusion of perspectives from regions with different socioeconomic backgrounds. It should be clearly stated that the inclusion of these hospitals in the CMEI sampling frame was not based on random or stratified sampling conducted by the research team for this specific study, but rather a non-probability sampling frame established by the CMEI on the basis of long-term data sharing agreements and the stability of data reporting by these medical institutions. The adoption of this non-probability frame was justified by two critical considerations: 1. data reliability: Hospitals in the frame undergo annual data validation by the CMEI, with a low data missing rate for key indicators, which meets the quality requirements for quantitative analysis of drug availability; and 2. policy relevance: The frame primarily includes secondary and tertiary public general hospitals, which are the main implementers of the NCDP policy, aligning with the study’s focus on evaluating NCDP implementation. To assess sample representativeness, the authors compared it with national data published in the China Health Statistics Yearbook (2019–2022), including: 1. quantitative representation: During the study period, the samples covered an average of 13.79% of the total number of public general hospitals, representing 8.38% of the total number of public hospitals, or 2.77% of the total number of hospitals nationwide in China; and 2. hierarchical distribution: The internal composition of the samples—with tertiary public general hospitals accounting for an average of 66.09% and secondary public general hospitals for 33.91%—is consistent with the policy attention and significant market share of tertiary hospitals in the promotion and use of NCDP drugs, thereby enhancing the reference value of this study’s results for policy evaluation. Although the non-randomness of the samples is a methodological limitation, it is important to clarify the boundaries of generalizability: the findings cannot be directly generalized to all hospital types in China (e.g., primary care institutions and small private hospitals with inadequate data systems). However, the samples’ comprehensive coverage of 31 provincial administrative regions, consistency with national benchmarks in economic development and hierarchical distribution, and reliable data quality can ensure their relevance for policy evaluation. Specifically, the results are generalizable to public general hospitals—the core implementers of the NCDP policy—across regions with diverse economic development levels, which aligns with the primary target of this study.

### Data analysis

#### Availability

In the 2008 WHO/HAI standard survey method, the drug availability rate, namely, the proportion of the institutions where a drug is available to all the institutions surveyed, is used to evaluate the availability of drugs. Some scholars have claimed that the applicability of this method in China is limited and that the drug procurement rate and drug availability rate in research institutions should be used instead to reflect drug availability in China (Lu et al., 2023; Li et al., 2018). The drug availability rate = the number of institutions that can provide the drugs surveyed/the total number of the institutions surveyed × 100%, and this measure emphasizes evaluating availability at the drug level. The drug procurement rate in research institutions = the number of the drug varieties surveyed accessible in the institutions/the total number of the drug varieties surveyed × 100%, and this measure focuses on evaluating availability at the research institution level. The overall drug procurement rate in a certain region is the median value of drug procurement rates in the region in question. As these evaluation methods are relatively comprehensive, the authors have drawn on them to investigate the availability of NCDP drugs. The availability of NCDP drugs was evaluated by reference to relevant studies conducted by other scholars (Dong et al., 2020; Li et al., 2018; Saeed et al., 2021), where 0 indicates not available, <30% very low availability, 30%–49% low availability, 50%–80% relatively high availability, and >80% high availability.

### Statistical analysis

The analysis was conducted by virtue of SPSS software (version 22.0; SPSS Inc., Chicago, IL, USA). Comparisons between two groups were performed via the Mann‒Whitney test, and comparisons among more than three groups were performed via the Kruskal‒Wallis test. Differences were considered to be statistically significant when P < 0.05.

## Results

### Availability rates of NCDP drugs

The results presented in Table 1 reveal as follows: 1. Differences in the average availability rates of the first to fifth batches of NCDP drugs among regions with different economic development levels were significant (P < 0.001), and the overall ranking in availability rates was developed regions, moderately developed regions, and less developed regions. A difference was observed in the average availability rate of different batches of NCDP drugs (P < 0.05). The average availability rate of each batch of NCDP drugs was relatively high in developed regions (from 53.34% to 70.42%). In moderately developed regions, with the exception of the fourth batch of NCDP drugs (47.30%), which exhibited low availability, the average availability rates pertaining to the remaining batches were relatively high (from 50.08% to 65.14%). In less developed regions, only the average availability rates of the first batch of “4 + 7” expansion and the second batch of NCDP drugs were relatively high (67.94% and 52.04%, respectively), whereas the rates of the remaining batches were low. Since the 11 cities included in the “4 + 7” pilot plan are not considered as less developed regions, the average availability rate of the first batch of the “4 + 7” pilot plan in less developed regions was unrecorded in the analysis. 2. The average availability rates of the first to fifth batches of NCDP drugs in developed regions, moderately developed regions and less developed regions all tended first to decrease and subsequently to increase.

**TABLE 1:** Availability rates of the five batches of NCDP drugs in developed, moderately developed, and less developed regions.

Batch	Developed regions		Moderately developed regions		Less developed regions		P-value
	Mean availability rate (%)	Evaluation	Mean availability rate (%)	Evaluation	Mean availability rate (%)	Evaluation	
The first batch of the “4 + 7” pilot plan	65.68	Relatively high	56.44	Relatively high	—	—	0.041
The first batch of “4 + 7” expansion	70.42	Relatively high	65.14	Relatively high	67.94	Relatively high	0.374
The second batch	63.84	Relatively high	58.88	Relatively high	52.04	Relatively high	0.051
The third batch	57.37	Relatively high	53.50	Relatively high	49.36	Low	0.228
The fourth batch	53.34	Relatively high	47.30	Low	44.46	Low	0.135
The fifth batch	54.42	Relatively high	50.08	Relatively high	45.78	Low	0.163
All batches	59.02	Relatively high	53.82	Relatively high	50.04	Relatively high	0.000
P-value	0.026		0.023		0.001		​

‘—’ indicates no procurement data available for this batch during the study period.

### Availability rates of different types of NCDP drugs

The results presented in Table 2 reveal as follows: 1. The average availability rates of all types of NCDP drugs in regions with different levels of economic development did not differ significantly. In general, the availability rates in developed regions were higher than those in moderately developed regions and less developed regions, and less developed regions were associated with the lowest rates. In developed regions, with the exceptions of drugs for the genitourinary system and sex hormones, and dermatologicals drugs, which featured low levels of availability, the other drug categories showed relatively high availability rates. In moderately developed regions, the average availability rate of dermatologicals drugs was very low; the average availability rates of respiratory system drugs (47.15%), nervous system drugs (47.79%), and drugs for the genitourinary system and sex hormones (44.53%) were low; and the average availability rates of other drug categories were relatively high. In less developed regions, the evaluation results indicated that low average availability rates were only concerning systemic anti-infective drugs and systemic hormonal preparations, excl. sex hormones and insulins, whereas the average availability rates of all other drug categories were comparable to those observed in moderately developed regions. 2. Among the five batches of NCDP drugs, the top five drug categories according to quantity-based rankings were systemic anti-infective drugs, drugs for alimentary tract and metabolism, nervous system drugs, cardiovascular system drugs, and antineoplastic and immunomodulating agents. However, differences were observed in the rankings of the average availability rate and the included quantities with regard to these five categories of drugs.

**TABLE 2:** Availability rates of different categories of NCDP drugs in developed, moderately developed and less developed regions.

Therapeutic category	Developed regions		Moderately developed regions		Less developed regions		P-value
	Mean availability rate (%)	Evaluation	Mean availability rate (%)	Evaluation	Mean availability rate (%)	Evaluation	
Drugs for alimentary tract and metabolism	67.08	Relatively high	58.08	Relatively high	52.01	Relatively high	0.030
Musculo-skeletal system drugs	63.60	Relatively high	58.15	Relatively high	54.20	Relatively high	0.504
Cardiovascular system drugs	63.42	Relatively high	58.67	Relatively high	58.25	Relatively high	0.443
Systemic anti-infective drugs	60.52	Relatively high	53.18	Relatively high	48.86	Low	0.064
Systemic hormonal preparations, excl. sex hormones and insulins	59.60	Relatively high	53.48	Relatively high	47.44	Low	0.368
Blood and blood-forming organ drugs	59.26	Relatively high	56.04	Relatively high	54.88	Relatively high	0.916
Others	56.59	Relatively high	69.46	Relatively high	57.39	Relatively high	0.180
Sensory organs drugs	56.48	Relatively high	56.30	Relatively high	53.38	Relatively high	0.700
Antineoplastic and immunomodulating agents	56.31	Relatively high	54.79	Relatively high	54.57	Relatively high	0.848
Respiratory system drugs	55.79	Relatively high	47.15	Low	45.65	Low	0.417
Nervous system drugs	51.18	Relatively high	47.79	Low	40.37	Low	0.149
Drugs for the genitourinary system and sex hormones	48.72	Low	44.53	Low	38.59	Low	0.719
Dermatologicals drugs	40.69	Low	27.85	Very low	22.44	Very low	0.368
P-value	0.288	​	0.513	​	0.307	​	​

### Procurement rates of NCDP drugs

**FIGURE 1:**
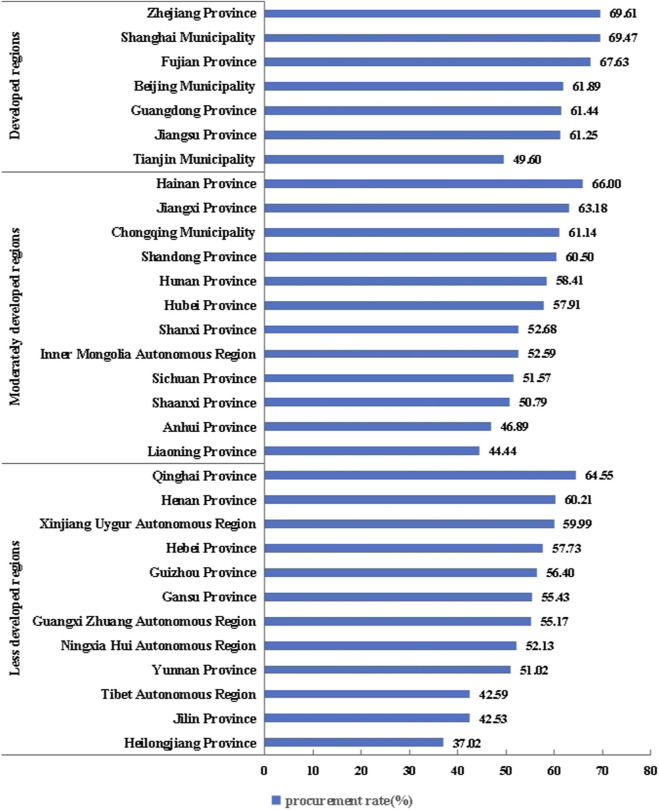
Procurement rates of the five batches of NCDP drugs in each provincial-level administrative region.

The results presented in Figure 1 and Table 3 indicate that 1. the procurement rates of the first to fifth batches of NCDP drugs in regions with different economic development levels differed significantly (P < 0.01) according to the ranking pattern of developed regions, moderately developed regions, and less developed regions. Significant differences were observed in the procurement rates of different batches of NCDP drugs across these regions (P < 0.001). In developed regions, the procurement rates of all batches of NCDP drugs exceeded 50%; in moderately developed regions, the procurement rates for the remaining batches of NCDP drugs exceeded 50%, with the exception of the fourth batch, which was less than 50%; and in less developed regions, only the procurement rates of the first batch of “4 + 7” expansion and the second batch of NCDP drugs exceeded 50%. The procurement rates of the first to the fifth batches of NCDP drugs in these regions all exhibited a trend of decreasing first and increasing subsequently. 2. Among developed regions, the top three regions in terms of the procurement rates of the five batches of NCDP drugs were Zhejiang Province, Shanghai Municipality and Fujian Province, and the region that exhibited procurement rates of less than 50% was Tianjin Municipality. Among moderately developed regions, the top three regions in terms of the procurement rates of the NCDP drugs were Hainan Province, Jiangxi Province and Chongqing Municipality, and the regions that exhibited procurement rates of less than 50% were Anhui Province and Liaoning Province. Among less developed regions, the top three regions in terms of the procurement rates of the five batches of NCDP drugs were Qinghai Province, Henan Province and Xinjiang Uygur Autonomous Region, whereas the regions that exhibited procurement rates of less than 50% included Tibet Autonomous Region, Jilin Province and Heilongjiang Province. However, in some provinces of moderately developed regions, the procurement rates of NCDP drugs were higher than those observed in some provinces in developed regions. For example, the procurement rates observed in Hainan Province and Jiangxi Province were higher than those observed in Beijing Municipality, Guangdong Province, Jiangsu Province, and Tianjin Municipality. Moreover, some less developed regions exhibited higher procurement rates regarding NCDP drugs than developed and moderately developed regions. For instance, Qinghai Province exhibited higher procurement rates than Beijing Municipality, Guangdong Province, Jiangsu Province, Tianjin Municipality, Jiangxi Province, Chongqing Municipality, Shandong Province, and Hunan Province.

**TABLE 3:** Procurement rates of the five batches of NCDP drugs in developed, moderately developed and less developed regions.

Batch	Developed regions	Moderately developed regions	Less developed regions	P-value
	Procurement rate (%)	Procurement rate (%)	Procurement rate (%)	
The first batch of the “4 + 7” pilot plan	76.00	64.00	—	0.000
The first batch of “4 + 7” expansion	74.68	71.11	66.56	0.005
The second batch	64.52	61.29	51.61	0.004
The third batch	58.34	52.12	48.79	0.000
The fourth batch	55.56	48.89	44.44	0.000
The fifth batch	60.71	50.43	49.11	0.000
All batches	64.97	57.97	52.10	0.000
P-value	0.000	0.000	0.000	​

‘—’ indicates no procurement data available for this batch during the study period.

## Discussion

The data obtained in this study reveal that the overall availability rate and procurement rate of NCDP drugs are characterized by a stepwise distribution pattern that “their rates in developed regions is higher than those in moderately developed regions which is also higher than those in less developed regions”. This pattern may be the result of the correlation between regional economic development and the level of medical technology (Ya-Qing et al., 2023). Typically, the higher the level of regional economic development is, the higher the level of the corresponding medical and health institutions will be; and thus, the more medical needs are present, the better the NCDP policy will be implemented in the corresponding area. Studies have indicated that the level of economic development is the core driver of global disparities in the availability of novel anti-cancer drugs. Higher economic development is associated with a greater variety of such drugs on the market and a shorter lag in their launch (Li M. et al., 2024). On the other hand, this pattern may be related to regional medical insurance policies and financial support. Medical insurance departments in more developed regions usually have sufficient funds for drug reimbursement, which can encourage healthcare institutions to purchase NCDP drugs actively. In contrast, medical insurance funds in less economically developed regions are relatively tight, which may impose certain restrictions on drug procurement and reimbursement. This characteristic also has similar corroboration at the international level. Studies focusing on drug accessibility in Brazil have clearly indicated that the long-term stability of drug funding is the core prerequisite for ensuring drug accessibility, while disadvantages in financial support—such as insufficient investment in the healthcare system and strained medical insurance funds—directly dampen the enthusiasm for drug supply and procurement (Ruas et al., 2025). In addition, this pattern may be associated with the location of drug distribution companies. In more developed regions, drug distribution companies are densely laid out, and the well-run infrastructure, including logistics infrastructure, is conducive to the efficient transportation and distribution of NCDP drugs to various healthcare institutions. In contrast, less economically developed regions accommodate fewer drug distribution companies whose coverage in these regions is limited. In this way, it leads to long drug distribution cycles and high costs, thereby affecting the availability of drugs. In response, relevant Chinese authorities have also explicitly stated that they will fully leverage the logistical network strengths of postal and express delivery enterprises to enhance medicine supply and guarantee capabilities at the grassroots and in remote areas. This provides crucial policy guidance for addressing the drug distribution bottlenecks in less developed regions (The State Council of The People’s Republic of China, 2025a; The State Council of The People’s Republic of China, 2025b).

This study also reveals the absence of a single linear correlation between regional economic development levels and the availability of NCDP drugs. In fact, differences are observed across regions. Among developed regions, Zhejiang Province, Shanghai Municipality, and Fujian Province have achieved remarkable results in the allocation of NCDP drugs. The sound medical infrastructure, sufficient financial support, efficient medical management systems, and mature and stable drug supply chain networks in these regions can provide solid guarantees for the implementation of the NCDP policy. For example, Zhejiang Province has innovatively developed a “cloud pharmacy” platform to optimize drug dispensing and supply processes by using digital means and improve the distribution efficiency and accessibility of NCDP drugs (Zhejiang Provincial Medical Security Bureau, 2020). The “sunshine procurement” mechanism implemented in Shanghai Municipality has enhanced the standardization and competitiveness of the procurement link, effectively reduced procurement costs, and accelerated the entry of NCDP drugs into healthcare institutions (Shanghai Municipal Medical Security Bureau, 2020). In addition, these regions have responded quickly to national policies, actively linked the status of NCDP completion with the ratings of healthcare institutions, and, through administrative assessments, strongly promoted the implementation of the NCDP policy. However, as a municipality directly under the central government, Tianjin features a procurement rate of NCDP drugs of less than 50%, thus highlighting the possible deviation of policy implementation in economically developed regions and supporting the statement that “an economic advantage does not equal policy efficacy”. Tertiary hospitals in Tianjin are highly concentrated. Some physicians have long relied on reference listed drugs (RLDs) or imported drugs, and thus had a low level of acceptance of NCDP drugs, especially generic drugs, which has affected the clinical promotion and use of NCDP drugs (Ningdu County People’s Government, 2025; Li et al., 2025). The prices of NCDP drugs have decreased significantly, which has compressed the profit margins of hospital pharmacies and impacted the revenue structure of hospitals, thereby reducing hospitals’ motivation to promote the use of NCDP drugs (Chen et al., 2021; Tianjin Municipal Medical Security Bureau, 2022; Jiang et al., 2022). Furthermore, the long-established drug distribution profit chain, consisting of an alliance between local pharmaceutical companies and traditional distributors, has hindered the smooth circulation of NCDP drugs (National Health Commission of the People’s Republic of China, 2021; Cui and Lyu, 2018). The supervision of the NCDP policy by local regulatory agencies is insufficient, and the lack of a strident constraint mechanism prevents the full implementation of the policy (Chang et al., 2024; Ningdu County People’s Government, 2025). Among moderately developed regions, Hainan Province, Jiangxi Province, and Chongqing Municipality have performed well in the allocation of state-purchased drugs, and their performance is even better than that observed in some developed regions. Hainan Province, with the advantages of the free trade port policy, has attracted a large number of medical resources to achieve an increase in medical resources and accelerate the introduction and promotion of NCDP drugs (Hainan Provincial People’s Government, 2021). In recent years, Jiangxi Province has vigorously promoted the development of a county-level medical community. Through the integration of the resources of healthcare institutions at all levels throughout the county and the consolidation of the connections and cooperation among healthcare institutions, the entire process of drug procurement, distribution and use has been optimized, and the accessibility of NCDP drugs has increased (Jiangxi Provincial Health Commission, 2021). Chongqing Municipality, by virtue of its advantages in administrative management and resource allocation, has exhibited an outstanding ability of NCDP management and is able to match the needs of healthcare institutions accurately, coordinate resources efficiently, and ensure the timely provision of NCDP drugs in sufficient quantities to all healthcare institutions (Chongqing Municipal Medical Security Bureau, 2022; Chongqing Municipal People’s Government, 2024). In contrast, Anhui and Liaoning provinces are characterized by low procurement rates for NCDP drugs. Anhui boasts a very large population base and a strong demand for medical resources, but its resource distribution is uneven. Healthcare institutions in remote areas are constrained by factors such as remote geographic locations, inconvenient transportation, and low levels of informatization, which may make it difficult to ensure that NCDP drugs are available in remote healthcare institutions (General Office of Anhui Provincial People’s Government, 2016; Anhui Provincial Health Commission, 2023). As an old industrial base, Liaoning is undergoing a critical period of industrial restructuring and upgrading, facing increased pressure from economic restructuring. Accordingly, medical reform may have a relatively low priority in terms of resource investment and policy promotion efforts. During the NCDP policy implementation process, the input of dedicated manpower, material resources, and financial resources may be insufficient, which in turn affects the coverage of NCDP drug procurement (Liaoning Provincial People’s Government, 2021; Ministry of Finance of the People’s Republic of China, 2019). Existing studies have lent empirical support to this argument from the dual perspectives of policy implementation and market supply. Based on a nationwide survey of 329 medical institutions, Zhang et al. (2023) verified significant regional disparities in the implementation of the NCDP policy, and identified that inadequate drug supply security in primary and remote areas is closely associated with imbalanced local resource investment, thereby corroborating that insufficient resource input may restrict the procurement coverage of NCDP drugs in old industrial bases such as Liaoning. From the perspective of market structures, Yang et al. (2024) further pointed out that under the volume-based procurement mechanism, small and medium-sized enterprises (SMEs) with disadvantaged resource endowments face intense competitive pressure. This may lead to dwindling numbers of market players and supply shortages, thus exacerbating inadequate procurement coverage. In summary, these two studies have demonstrated that insufficient investment in dedicated resources impairs policy implementation efficiency and market supply capacity, ultimately exerting an adverse impact on NCDP procurement coverage. Among less developed regions, Qinghai Province, Henan Province, and Xinjiang Uygur Autonomous Region are characterized by relatively high procurement rates, which are even higher than those observed in some developed and moderately developed regions. Qinghai and Xinjiang have benefited from central transfer payments and construction aid policies, such as special medical projects aimed at supporting the Western Development. These have, to a certain extent, alleviated the shortage of medical resources resulting from lagging economic development and enhanced the ability of relevant actors in these regions to procure and supply NCDP drugs. However, the long-term sustainability of the policy requires further evaluation (Qinghai Provincial Health Commission, 2024; The State Council Information Office of the People’s Republic of China, 2022; The State Council Information Office of the People’s Republic of China, 2020). On the basis of the county medical community model, Henan has integrated medical resources within the region, used the strategy of “trading volume for price” to motivate healthcare institutions to procure NCDP drugs, and promoted the extensive allocation of drugs to healthcare institutions at all levels (Henan Provincial Health Commission, 2021a; 2021b). However, the procurement rates observed in Tibet, Jilin Province and Heilongjiang Province are low. The plateau environment of Tibet and the cold climates of Heilongjiang and Jilin contribute to the difficulty and high costs of drug distribution, making it difficult to guarantee timely drug distribution, and decreasing the willingness of pharmaceutical companies to supply drugs (Sina Finance, 2024a; Sina News, 2024; Sina Finance, 2024b). In Tibet, the limited number of sample hospitals included in this study may contribute to the low procurement rate of NCDP drugs.

The results of this study reveal that in developed, moderately developed and less developed regions, the availability of all types of NCDP drugs tends to decrease generally. This phenomenon may be accounted for by the increased diversity of disease types present in developed regions (Oldfield et al., 2025). The availability of different categories of NCDP drugs varies across regions with different economic development levels, and the factors influencing this situation are complex. In addition to the incidence and treatment cycle of the disease, the therapeutic status of the drug in terms of clinical diagnosis and treatment, and public health emergencies, the attributes of the drug itself cannot be ignored. These attributes include whether a drug is included in the national basic medical insurance policies (and whether the payment range is restricted in the remarks), whether the drug is listed as a key monitored drug, and whether it is used for special populations (Mao et al., 2022; Xiong et al., 2024). Notably, the availability of dermatologicals drugs is the lowest among all types of drugs. It can be mainly attributed to the facts that this drug category falls under specialty medications, and that patients with dermatosis usually visit specialized hospitals to receive medical treatment (Danielsen et al., 2025). Therefore, specialized hospitals were excluded from this study, as the China CMEI contains only a small number of such hospitals with an uneven national distribution. Consequently, this study was limited to general hospitals, which may explain the poor availability of dermatologicals drugs observed. The study also reveals that, among the five batches of NCDP drugs, the top five drug categories in terms of quantity are similar to the drug categories with the highest sales volume in the chemical drug market in China’s public healthcare institutions from 2020 to 2022 (China Commerce Industry Research Institute, 2021; China Commerce Industry Research Institute, 2022). This finding indicates that NCDP drugs largely meet the actual needs of the Chinese pharmaceutical market. However, the results of this study also reveal certain differences in the rankings of all types of NCDP drugs in terms of availability and inclusion quantity, including notably significant differences in the use of systemic anti-infective drugs and nervous system drugs. Systemic anti-infective drugs rank first in inclusion quantity but only upper-middle in overall availability across regions with different economic development levels; nervous system drugs rank third in inclusion quantity but are consistently among the lowest in overall availability across these regions. On the one hand, the local NCDP process may feature repeated purchases of drugs with similar indications (Zang et al., 2022), thus affecting the ranking of drug availability in this context. On the other hand, systemic anti-infective drugs are subjected to special controls, and their use and procurement are highly restricted, whereas most nervous system drugs are used for special populations. A large deviation is also evident between the availability and the inclusion quantity rankings of these two types of drugs.

The results of the study also indicate that the early batches of NCDP drugs tend to exhibit better availability than the later batches. This finding reveals that the market availability status of each batch of NCDP drugs is correlated with the number of varieties and the time of market availability. Specifically, the fewer varieties are included, the easier it will be to market them; furthermore, the longer the market cycle is, the greater the extent to which the NCDP policy will be implemented. In addition, the results of this study reveal that in developed, moderately developed and less developed regions, the availability of NCDP drugs tend to increase to some extent in the fifth batch. This finding may be explained by the standardization and institutionalization of the NCDP policy and the gradual increase in the types and dosage forms covered by the policy. These conclusions are consistent with the results of related research conducted by the authors on hospitals at different levels (Lu et al., 2023).

In summary, although the availability of NCDP drugs improved following the implementation of the NCDP policy, the policy can go even further. Inspired by the results of the study, the following suggestions are proposed: 1. Regionally differentiated policy control. In developed regions, assessments of the use proportion of NCDP drugs in tertiary healthcare institutions should be strengthened; the allocation of NCDP drugs should be linked to the disbursement of medical insurance funds; and an incentive mechanism should be established to replace RLDs with NCDP drugs so as to encourage physicians to prioritize NCDP varieties. In less developed regions, firstly, the drug distribution networks should be optimized, and efforts to construct logistics distribution centres should be increased in areas with inconvenient transportation and weak logistics infrastructure. Secondly, drug reserve mechanisms should be established. In accordance with the geographic environment, disease spectrum and area-specific demand for medication, a reasonable drug reserve catalogue and reserve quantity standards should be set up to address possible fluctuations in the drug supply. Thirdly, governmental departments can increase the investment and focus of medical insurance funds appropriately to reduce the financial burdens suffered by patients and improve the extent to which both patients and healthcare institutions understand the NCDP policy. Fourthly, the medical service capabilities of healthcare institutions should be enhanced by providing telemedicine and peer support to prevent the insufficient use of NCDP drugs limited by medical technology. Fifthly, healthcare institutions should be allowed to make joint purchases across regions. 2. Improving the implementation motivation of healthcare institutions. A scientific drug procurement volume forecast mechanism should be established to provide accurate predictions of the NCDP drugs purchase. The use of NCDP drugs should be included in the process of calculating the weights of Diagnosis-Related Groups/Diagnosis-Intervention Packet (DRG/DIP) disease groups to ensure that healthcare institutions can retain savings from reduced drug expenditures. Real-world studies on the efficacy of NCDP drugs should be conducted with the aim of reassuring both doctors and patients on the basis of data-based evidence. The use proportion of NCDP drugs should be included in the performance assessments of physicians. Delivery enterprises with good reputations and strong delivery capabilities should be chosen, and clear delivery contracts should be signed, including terms regarding aspects such as delivery time, frequency, and quality. 3. Strengthening supply chain security. In terms of enterprise incentives and constraints, a “supply rate–market share” linkage mechanism should be implemented for enterprises that win bids pertaining to national centralized procurement. If their supply rates meet the relevant standard, these enterprises should get priority in participating in the next round of the NCDP. Government departments should strengthen legal supervision, impose severe penalties on enterprises that fail to supply drugs in accordance with the corresponding contracts, and establish a regular communication mechanism with enterprises, ensuring awareness of the production status, raw material supply, production capacity and other aspects. Accordingly, the problems that affect the drug supply can be discovered and timely measures can be taken to address them. Enterprises should formulate scientific and reasonable production plans according to the requirements of the NCDP contracts to ensure that drug production can meet the corresponding demand. Moreover, enterprises should formulate drug supply contingency plans and countermeasures in advance in case of possible production interruptions, raw material shortages, transportation obstructions and other emergencies. In terms of logistics distribution, with the support of smart logistics, the promotion of unmanned aerial vehicle (UAV) delivery and smart medicine cabinets in remote areas can reduce the cost of terminal distribution. Besides, a hierarchical approach to the management of distribution companies can be implemented, and medical insurance designated points can give priority to companies that complete delivery tasks in remote areas.

This study also has certain limitations. First, the hospital samples in this study were sourced from fixed monitoring sites in the CMEI database, which constitutes a non-probability sampling method. Although this sampling frame offers extensive geographical coverage and the hierarchical distribution of hospitals reflects key market characteristics, the non-random nature of the sampling made it impossible to completely exclude selection bias. Therefore, caution should be exercised when generalizing the study results to all hospitals nationwide. Future research could adopt mixed sampling methods (combining the CMEI frame with stratified random sampling of primary care institutions) to further improve generalizability. Second, the research institutions covered by this study were exclusively general hospitals, and specialized hospitals and retail pharmacies were not included in the study, which may result in an incomplete sample. Third, in some regions, the number of healthcare institutions surveyed was limited, and the limited sample size may affect the representativeness of the study results. Fourth, during the time interval covered by this study, the procurement cycle of some drugs ended, whereas the procurement cycle of other drugs had not yet been completed. This temporal inconsistency may have interfered with judgements regarding the overall availability of drugs. Fifth, in light of the wide area of China and the large number of batches and types of NCDP drugs, the price levels and affordability of NCDP drugs were not investigated, despite the fact that both of these factors have tremendous impacts on drug accessibility. Future research may investigate these issues to produce a more comprehensive and accurate evaluation of the accessibility of NCDP drugs.

## Conclusion

Overall, this study has revealed that the more economically developed the regions are, the more available NCDP drugs tend to be. It has also revealed differences in the supply capabilities of NCDP drugs in regions with different economic development levels in China, thus highlighting regional imbalances pertaining to policy implementation. In terms of the availability of NCDP drugs, developed regions maintained their dominant position, but they also exhibited deviations in terms of policy implementation. Moderately developed regions were characterized by internal differences, and the availability of NCDP drugs in some regions approached or even exceeded the corresponding levels in developed regions, whereas other regions converged with less developed regions. Although less developed regions face multiple resource constraints, local breakthroughs can be achieved on the basis of innovation and collaboration. This finding can serve as an important reference for efforts to optimize the differentiated implementation of the NCDP policy. In essence, the results of this study reflect the distribution of medical resources, policy implementation and the interactions among various interests. In the future, through the combination of a “refined policy design + intelligent supply chain + long-acting incentive mechanism”, the balanced implementation of the NCDP policy among regions should be promoted with the aim of achieving synergy among patient accessibility, healthcare institutional sustainability, and enterprise vitality.

## Data Availability

The original contributions presented in the study are included in the article/Supplementary Material, further inquiries can be directed to the corresponding author.
