# Supplementary material for: A study on the availability of national centralized drug procurement in regions with different levels of economic development: an investigation and analysis of 31 provincial-level administrative regions in China
Source: Front Pharmacol. 2026 Jan 28;17:1652715. doi: 10.3389/fphar.2026.1652715 (PMC12890630; doi:10.3389/fphar.2026.1652715)
Supplement: Supplementary file 1 [file Supplementaryfile1.docx]

**SUPPLEMENTAL FIGURES AND TABLES**

**Supplementary Table 1** Overview of information regarding the procurement cycle of the five batches of NCDP drugs

| Batch | The total number of NCDP drugs Varieties (Units) | The procurement cycle（The number of NCDP drugs Varieties during the procurement cycle） |
| --- | --- | --- |
| The first batch of the "4+7" pilot plan | 25 | 01/04/2019–31/03/2020 (25)* |
| The first batch of “4+7” expansion | 25 | 23/12/2019–22/12/2020 (11)*  +23/12/2019–22/12/2021 (14)* |
| The second batch | 32 | 27/04/2020–26/04/2021 (1)*  +27/04/2020–26/04/2022 (20)*  +27/04/2020–26/04/2022 (11) |
| The third batch | 55 | 20/10/2020–19/10/2021 (22)*  +20/10/2020–19/10/2022 (8)*  +20/10/2020–31/12/2022 (25) |
| The fourth batch | 45 | 27/04/2021–26/04/2022 (15)*  +27/04/2021–31/12/2022 (30) |
| The fifth batch | 61 | 01/11/2021–31/10/2022 (13)*  +01/11/2021–31/12/2022 (48) |

Note: '*' indicates that the procurement cycle for NCDP drugs has ended.

**Supplementary Table 2** Distribution of the 31 provincial-level administrative regions with different levels of economic development

| Regions by economic development level | Provincial-level administrative regions |
| --- | --- |
| Developed regions | Beijing Municipality; Shanghai Municipality; Jiangsu Province; Fujian Province, Zhejiang Province; Tianjin Municipality; Guangdong Province |
| Moderately developed regions | Chongqing Municipality; Hubei Province; Inner Mongolia Autonomous Region; Shandong Province; Shaanxi Province; Anhui Province; Hunan Province; Jiangxi Province; Liaoning Province; Shanxi Province; Sichuan Province; Hainan Province |
| Less developed regions | Ningxia Hui Autonomous Region; Xinjiang Uygur Autonomous Region; Henan Province; Yunnan Province; Qinghai Province; Jilin Province; Tibet Autonomous Region; Hebei Province; Guizhou Province; Guangxi Zhuang Autonomous Region; Heilongjiang Province; Gansu Province |

**Supplementary Table 3** Distribution of hospital samples in regions with different levels of economic development

| Period | Number of public general hospitals in developed regions (Units) | Number of public general hospitals in moderately developed regions (Units) | Number of public general hospitals in less developed regions (Units) | Total (Units) |
| --- | --- | --- | --- | --- |
| 2019–2020 | 261 | 387 | 320 | 968 |
| 2019–2021 | 249 | 370 | 313 | 932 |
| 2020–2021 | 262 | 388 | 334 | 984 |
| 2020–2022 | 255 | 371 | 321 | 947 |
| 2021–2022 | 272 | 393 | 352 | 1017 |

**Supplementary Table 4** Distribution of the number of varieties of the five batches of NCDP drugs in different categories

| Therapeutic category | Drug varieties | The first batch | The second batch | The third batch | The fourth batch | The fifth batch | Total |
| --- | --- | --- | --- | --- | --- | --- | --- |
| Systemic anti-infective drugs | Systemic antibacterials; Antituberculars; Systemic antifungals; Systemic antivirals | 3 | 10 | 8 | 6 | 12 | 39 |
| Drugs for alimentary tract and metabolism | Anti-acid-related disorder agents; Gastroprokinetics; Antidiarrheals; Antiemetics; Laxatives; Vitamins; Antidiabetics; Mineral supplements | 1 | 4 | 8 | 9 | 11 | 33 |
| Nervous system drugs | Antidepressants; Antipsychotics; Antiepileptics; Anti-parkinson agents; Anti-dementia drugs; Hypnotics and sedatives; Antipyretics and analgesics;  Anesthetics | 6 | 2 | 10 | 8 | 3 | 29 |
| Cardiovascular system drugs | Renin-angiotensin system agents; Cardiac therapy agents; Antihypertensives; Lipid regulators; Peripheral vasodilators; Calcium channel blockers; Beta blockers | 9 | 7 | 4 | 4 | 5 | 29 |
| Antineoplastic and immunomodulating agents | Antineoplastics; Endocrine therapy agents; Immunosuppressants; Immunostimulants | 3 | 3 | 5 | 3 | 8 | 22 |
| Respiratory system drugs | Cough and cold preparations; Drugs for obstructive airway diseases; Antihistamines | 1 | 2 | 5 | 4 | 6 | 18 |
| Blood and blood-forming organ drugs | Antithrombotics; Antianaemics; Blood substitutes and perfusion solutions | 1 | 0 | 3 | 2 | 8 | 14 |
| Musculo-skeletal system drugs | Anti-inflammatory and antirheumatic drugs; Muscle relaxants; Antigout agents | 1 | 1 | 6 | 4 | 1 | 13 |
| Drugs for the genitourinary system and sex hormones | Urinary system drugs; Sex hormones; Oxytocics | 0 | 3 | 5 | 0 | 3 | 11 |
| Sensory organs drugs | Ophthalmologicals | 0 | 0 | 1 | 3 | 0 | 4 |
| Others | Contrast media; Diagnostic agents | 0 | 0 | 0 | 0 | 3 | 3 |
| Dermatologicals drugs | Dermatological antifungals | 0 | 0 | 0 | 1 | 0 | 1 |
| Systemic hormonal preparations, excl. sex hormones and insulins | Calcium homeostasis regulators | 0 | 0 | 0 | 0 | 1 | 1 |

**Supplementary Figure 1** Distribution of the surveyed public general hospitals in each provincial administrative region
